# Gut–lung axis in allergic rhinitis: microbial dysbiosis and therapeutic strategies

**DOI:** 10.3389/fmicb.2025.1654997

**Published:** 2025-12-12

**Authors:** WeiKeng Yang, Hui Wu, Xiuyun Li, Zhi Wan, Wei Kong, Congfu Huang

**Affiliations:** 1 The Second Affiliated Hospital, School of Medicine, The Chinese University of Hong Kong, Shenzhen & Longgang District People’s Hospital of Shenzhen, Shenzhen, Guangdong, China; 2 Child Healthcare Department, Maternal and Child Health Hospital of PanYu District (Hexian Memorial Hospital of PanYu District), Guangzhou, China; 3 Department of Pediatrics, Longgang District Maternity and Child Healthcare Hospital of Shenzhen City (Longgang Maternity and Child Institute of Shantou University Medical College), Medical Research Institute of Maternal and Child, Shenzhen, Guangdong, China

**Keywords:** allergic rhinitis, gut microbiota, gut–lung axis, short-chain fatty acids, probiotics, immune tolerance

## Abstract

**Background:**

Allergic rhinitis (AR) affects an estimated 10%–30% of people worldwide and places a significant burden on both health and healthcare systems. Recent research suggests that imbalances in the gut microbiota may contribute to the development of AR by disrupting immune regulation along the gut–lung axis. However, these insights have yet to be fully translated into clinical practice.

**Methods:**

We performed a systematic review of studies published between 2010 and 2025, including clinical research, animal experiments, and multi-omics analyses, retrieved from PubMed, Web of Science, Embase, Cochrane, CNKI, and Wanfang databases. The review aimed to evaluate immune mechanisms mediated by the gut microbiota and assess microbiota-targeted interventions in AR.

**Results:**

Patients with AR consistently show reduced fecal butyrate levels, with several studies reporting significant declines, alongside elevated serum IgE concentrations. These changes are closely linked to gut dysbiosis, characterized by reduced abundance of *Faecalibacterium* and imbalances in the Bacteroidetes/Firmicutes ratio. Dysbiosis appears to drive activation of the aryl hydrocarbon receptor (AhR) pathway, evidenced by a 1.5-fold increase in the kynurenine/tryptophan ratio (*p* < 0.05), and contributes to impaired regulatory T-cell function. Experimental evidence supports these associations: in murine models, fecal microbiota transplantation (FMT) reduced nasal IL-13 levels by as much as 60% in one study. In human trials, probiotic supplementation, particularly with *Clostridium butyricum*, was linked to reductions in serum IgE in some cohorts. Integration of multi-omics datasets further reveals conserved mechanisms, including butyrate-mediated histone deacetylase inhibition and vagus nerve–dependent suppression of mast cell activity. Moreover, combinatorial approaches, such as combining probiotics with FXR agonists, have yielded significant improvements in preclinical models, notably reducing nasal symptom scores.

**Conclusion:**

Gut dysbiosis contributes to the development of AR by disrupting immune–metabolic pathways along the gut–lung axis. Microbiota-targeted interventions hold promise for both the prevention and management of AR, especially in pediatric populations. To achieve long-term impact, public health strategies that combine dietary modifications with measures to reduce air pollution are urgently needed.

## 1 Introduction

Allergic rhinitis (AR) is an IgE-mediated inflammatory disease affecting 10%–30% of the global population and contributing to a substantial socioeconomic burden, with annual healthcare expenditures estimated to exceed $20 billion (Mims, 2014; Licari et al., 2023). Current treatment options, including intranasal corticosteroids, antihistamines, and allergen immunotherapy, primarily provide symptomatic relief without correcting the underlying immune dysregulation (Ponda et al., 2023; Sousa-Pinto et al., 2024). Importantly, up to 45% of individuals with AR go on to develop comorbid asthma, though the mechanistic pathways linking these conditions remain incompletely understood (Sousa-Pinto et al., 2024).

Emerging evidence suggests that gut microbiota dysbiosis plays a central role in the pathogenesis of AR through the gut–lung axis (Trompette et al., 2014; Mann et al., 2024). Short-chain fatty acids (SCFAs), especially butyrate, support regulatory T-cell (Treg) differentiation and attenuate Th2-driven inflammation by activating GPR43/GPR109A signaling pathways and inhibiting histone deacetylases (HDACs) (Kim, 2023; Liu et al., 2025). Clinical studies have shown that patients with AR exhibit a reduced abundance of Faecalibacterium and altered Bacteroidetes-to-Firmicutes ratios, both of which are associated with higher serum IgE levels and increased nasal eosinophilia (Liu et al., 2020; Zhou et al., 2021; Zhang et al., 2023). However, several critical gaps remain: (1) limited causal evidence derived from human cohort studies; (2) ethnic bias in existing data, with a predominance of Asian and European populations; and (3) insufficient application of multi-omics approaches to uncover patient-specific biomarkers.

This review seeks to address these gaps by: (1) synthesizing causal evidence that connects gut dysbiosis, SCFA depletion, and Th2 polarization across both clinical and preclinical models; (2) evaluating the efficacy and underlying mechanisms of microbiota-targeted interventions, including probiotics and FMT, in diverse patient populations; and (3) proposing an AI-driven framework to advance precision management of AR. In contrast to previous reviews that have primarily examined dietary fiber-derived SCFAs in asthma models (e.g., Trompette et al., 2014), AhR signaling in autoimmunity (Rosser et al., 2020), or the kynurenine pathway in allergy (Van der Leek et al., 2017) more broadly, this work introduces, for the first time, a comprehensive framework of the gut–lung axis specifically tailored to AR pathogenesis. By integrating multi-omics approaches, including metagenomics, metabolomics, and immunomics, with rigorous cross-species validation in both human cohorts and murine models, this review aims to provide a unified, mechanistic perspective on AR. This integrated framework underscores the pivotal contributions of three interconnected mechanisms in AR: dysbiosis-associated SCFA depletion, exemplified by reduced *Faecalibacterium* abundance; altered tryptophan metabolism leading to aberrant AhR activation, reflected in an elevated kynurenine-to-tryptophan ratio; and emerging neuroimmune interactions, such as vagus nerve-mediated suppression of mast cell activity. By synthesizing these multi-omics insights, this review advances the gut–lung axis paradigm in AR and provides a foundation for the development of mechanism-driven, microbiota-targeted therapeutic strategies.

This review introduces a novel and comprehensive framework that systematically integrates multi-omics evidence, including metagenomics, metabolomics, and immunomics, with rigorous cross-species validation spanning human cohorts and murine models. Through this approach, it delineates a mechanistic connection between gut dysbiosis and the pathogenesis of AR, thereby refining and advancing the current understanding of the gut–lung axis.

## 2 Methods

This systematic review adhered to the Preferred Reporting Items for Systematic Reviews and Meta-Analyses (PRISMA) guidelines and was prospectively registered in PROSPERO (CRD1061058).

### 2.1 Literature search and screening

Databases (PubMed, Web of Science, Embase, Cochrane, CNKI, Wanfang) were systematically searched for clinical, animal, and mechanistic studies published between January 2010 and May 2025 from inception until May 31, 2025. Preprints indexed in medRxiv and bioRxiv were included to capture the emerging evidence. All cited preprints are explicitly identified as such in the reference list. Search terms combined the following three domains:

1) Allergic rhinitis: “IgE-mediated rhinitis,” “seasonal rhinitis”;
2) Gut microbiota: “Dysbiosis,” “butyrate-producing bacteria”;
3) Interventions: “Probiotics,” “fecal microbiota transplantation (FMT).”

The PRISMA flowchart (Supplementary Figure 1) details the screening steps: 2,358 records were identified; 867 were excluded during title/abstract screening; 117 full-text studies met the inclusion criteria (68 clinical, 32 animal, 17 mechanistic).

### 2.2 Inclusion/exclusion criteria

Inclusion criteria: (1) Studies analyzing interactions between gut microbiota dysbiosis and AR pathogenesis; (2) Measurements of microbial metabolites (i.e., SCFAs, tryptophan) and immune markers (i.e., IgE, Treg/Th2 ratios); (3) Microbiota-targeted interventions (such as probiotics, FMT, and prebiotics).

Exclusion criteria: Non-AR rhinitis, case reports, duplicates, or uncontrolled studies (sample size < 20).

### 2.3 Data extraction and quality assessment

Two independent reviewers extracted data (agreement *κ* = 0.85). The study quality was assessed using (1) Observational studies: Newcastle–Ottawa Scale; (2) RCTs: Cochrane Risk of Bias Tool 2.0.

Publication bias was evaluated via funnel plots and Egger’s test; asymmetry was adjusted using the trim-and-fill method.

Supplementary Tables 1, 2 provided full exclusion rationales and global prevalence data, respectively.

### 2.4 Data synthesis and integration

1) Meta-analysis: Random-effects models (RevMan 5.4) calculated the standardized mean differences (SMDs) with 95% CIs. Heterogeneity (I^2^ > 50%) was addressed by using the DerSimonian–Laird method. Sensitivity analyses employed leave-one-out validation.
2) Stratified synthesis: Clinical and animal studies were analyzed separately and then integrated thematically. The subgroup analyses included: (1) age (<18/≥18 years); (2) region (Asia/Europe/North America); (3) intervention type (probiotics/FMT/combinatorial).
3) Multi-omics integration: Metagenomic, metabolomic, and immunomic data were mapped to pathways (e.g., SCFA-GPR43, AhR-Treg) using QIIME 2 and MetaboAnalyst 5.0. Bonferroni correction applied for metabolomics.

### 2.5 Ethical statement

This review employed publicly available published data; no new human/animal experiments were conducted. All cited studies complied with the Declaration of Helsinki and obtained ethics approval. All cited studies, including those indexed up to May 2025, complied with the Declaration of Helsinki and were derived from peer-reviewed publications or indexed preprints (bioRxiv/medRxiv).

## 3 Evidence synthesis: gut dysbiosis as a hallmark of allergic rhinitis

### 3.1 Epidemiological characteristics

#### 3.1.1 Global prevalence

AR has a global prevalence of 10%–20%, albeit there are significant regional and age differences (Supplementary Table 2). For instance, the prevalence of AR among children in China ranges from 10.8 to 21.1%, with higher rates in the urban areas, potentially due to the greater exposure to airborne allergens and pollutants (Wang et al., 2016; Subspecialty Group of Rhinology, Editorial Board of Chinese Journal of Otorhinolaryngology Head and Neck Surgery, and Subspecialty Groups of Rhinology and Pediatrics, Society of Otorhinolaryngology Head and Neck Surgery, Chinese Medical Association, 2022). In Iran, the prevalence among children was as high as 24.3%, closely related to environmental factors such as exposure to dust mites and air pollution (Kalmarzi et al., 2020). In some parts of Europe, such as Poland, local AR (LAR) accounted for 17.4% of AR patients, and its diagnosis relied on nasal mucosa provocation tests. The data available on allergic diseases in Africa are limited. According to a longitudinal study conducted by Zar et al. (2007), the prevalence of AR among South African adolescents aged 13–14 years was reported to be 20.2 and 25.4% during the period from 1995 to 2002. However, these estimates were derived from studies with relatively small sample sizes and incomplete regional representation, which may have affected their generalizability. These regional differences may be attributed to non-unified diagnostic criteria (e.g., LAR diagnosis is reliant on invasive tests), the synergistic effects of environmental pollutants (such as PM2.5 and volatile organic compounds) not being fully assessed, and population coverage bias (e.g., lack of data on the elderly and indigenous populations in Africa and South America).

#### 3.1.2 Limitations of epidemiological studies

1) Nonuniform diagnostic criteria: The diagnosis of LAR relies on nasal mucosa provocation tests, which are complex to perform and lack standardized procedures, influencing potential misdiagnoses or missed diagnoses (Bozek et al., 2019; Hoang et al., 2022).
2) Insufficient research on environmental factors: Most epidemiological studies fail to systematically incorporate the synergistic effects of environmental pollutants (e.g., PM2.5 and volatile organic compounds) on AR incidence, especially the long-term effects of urbanization-related air pollution, which have not been fully assessed so far (Li et al., 2022; Xu et al., 2023).
3) Population coverage bias: Data on the elderly and specific ethnic groups (e.g., African and South American indigenous populations) are still lacking, and most of the existing conclusions are based on populations from Europe, America, and East Asia (Licari et al., 2023; Xu et al., 2023).
4) Weak analysis of comorbidity associations: The mechanisms underlying the comorbidities of AR with asthma and atopic dermatitis are under-researched, lacking cross-regional, multicenter prospective cohort studies for validation (Shen et al., 2019; Testa et al., 2020).

### 3.2 Limitations of current treatments

#### 3.2.1 Intranasal corticosteroids

Intranasal corticosteroids (e.g., mometasone furoate and fluticasone propionate) are first-line treatments for AR, which effectively relieve nasal congestion and inflammation. However, long-term use may lead to nasal mucosal atrophy, epistaxis, and drug-induced rhinitis. The long-term use of nasal corticosteroids may cause adverse effects such as nasal mucosal atrophy, with some studies even reporting incidence rates of 10%–15% and some patients experiencing symptom rebound after discontinuing the medication (Bousquet et al., 2020; Sousa-Pinto et al., 2024). In addition, their regulatory effects on comorbidities (e.g., asthma) are limited, with only a partial improvement in the lower respiratory symptoms (Shen et al., 2019).

#### 3.2.2 Antihistamines

Second-generation oral or intranasal antihistamines (e.g., loratadine and azelastine) can rapidly relieve nasal itching and sneezing, but they are less effective in controlling nasal congestion and may induce drug resistance with long-term use. With respect to nasal congestion symptoms, approximately 30% of the AR patients demonstrate a poor response to monotherapy with antihistamines and require the combination of leukotriene receptor antagonists (Seresirikachorn et al., 2019). Moreover, antihistamines have a weak regulatory effect on Th2-immune imbalance and cannot block disease progression (Ponda et al., 2023).

#### 3.2.3 Leukotriene receptor antagonists

Leukotriene receptor antagonists (e.g., montelukast) relieve symptoms by inhibiting leukotriene-mediated inflammation, but their efficacy differs significantly among individuals. The improvement rate in nasal congestion symptoms caused by leukotriene receptor antagonists (such as Montelukast) is approximately 40%, albeit this improvement may be accompanied by neurological and psychiatric side-effects such as insomnia (5%) (Krishnamoorthy et al., 2020). Moreover, these drugs do not significantly improve the skin symptoms of patients with comorbid atopic dermatitis (Shen et al., 2019).

#### 3.2.4 Immunotherapy (desensitization therapy)

Immunotherapy requires a duration of 3–5 years; albeit, in some studies (such as in studies conducted in resource-limited areas), patient compliance has been <40% (Ponda et al., 2023). Approximately 5%–15% of patients may experience systemic adverse reactions (such as anaphylactic shock), although the proportion of drug discontinuation remains 3%–10%, whereas sublingual immunotherapy (SLIT) offers safety advantages (such as a discontinuation rate <5%) (Shamji et al., 2022; Creticos et al., 2024). Furthermore, the efficacy of immunotherapy is limited in patients with multiple sensitizations, as it has insufficient regulatory effects on established Th2-inflammatory memory (Creticos et al., 2024).

#### 3.2.5 Surgical intervention

Surgical procedures (e.g., radiofrequency ablation of the inferior turbinate) are suitable for treating intractable nasal congestion, but have a postoperative recurrence rate as high as 30% and may damage the nasal mucosal function, thereby exacerbating nasal dryness (Safia et al., 2024). Surgery cannot correct an immune imbalance and cause no improvement in systemic allergic symptoms (e.g., eye itching) (Creticos et al., 2024).

#### 3.2.6 Insufficient regulation of comorbidities by traditional therapies

Approximately 45% of AR patients have comorbid asthma, although intranasal corticosteroids only slightly reduce the frequency of asthma exacerbations and cause limited improvement in airway hyperresponsiveness (Shen et al., 2019). Antihistamines and montelukast have no significant therapeutic effects on itching or eczema in patients with comorbid atopic dermatitis, which highlights the limitations of the conventional therapies in systemic immune regulation (Ponda et al., 2023).

## 4 AR and immune dysfunction

### 4.1 Overactivation of Th2-immune responses

The core pathological mechanism of AR is the abnormal activation of Th2-immune responses, which can be characterized by the increased secretion of cytokines such as IL-4, IL-5, and IL-13; these cytokines drive eosinophilic infiltration and IgE-mediated inflammatory cascades (Su et al., 2023). IL-4 promotes the class-switching of B cells to produce allergen-specific IgE, whereas IL-5 and IL-13 enhance eosinophil differentiation and mucus hypersecretion, thereby exacerbating nasal mucosal edema and airway hyperresponsiveness (Su et al., 2023). Past studies have demonstrated that the proportion of Th2 cells in the nasal mucosa of AR patients was significantly elevated and that their activity was positively correlated with the serum IgE levels (Guo et al., 2024). In addition, gut microbiota dysbiosis reduces SCFA levels, further weakening the inhibition of Th2 responses and creating a vicious cycle of “gut–lung axis” immune imbalance (Trompette et al., 2014; Dong et al., 2024).

### 4.2 Impaired Treg function and defective immune tolerance

Treg cells maintain immune tolerance by secreting IL-10 and TGF-*β*, but in AR patients, Treg cell number and function are compromised. Clinical studies have demonstrated that the proportion of Tregs in the peripheral blood of AR patients was decreased by approximately 30% when compared with that in healthy controls and that their ability to suppress Th2 inflammation was weakened (*p* < 0.05) (Shi et al., 2011). Mechanistically, a decrease in the butyrate levels caused by gut microbiota dysbiosis inhibits HDAC activity, reducing acetylation of *Foxp3* and thereby weakening the stability and function of Treg cells (Eshleman et al., 2024). Past studies in animal models suggest that supplementation with *Clostridium butyricum* can increase the Treg ratio (by up to 40% in some studies) and inhibit allergic inflammation (Kim et al., 2019). These promising preclinical findings warrant further investigation in human trials. In the ovalbumin (OVA)-induced AR mouse model, intervention with bifidobacteria significantly inhibited nasal mucosal inflammation, with a 40% increase in the Treg proportion when compared with that in the control group (*p* < 0.01) (Kim et al., 2019).

### 4.3 Mucosal barrier disruption and allergen translocation

Damage to the nasal and gut mucosal barriers is a critical component of AR pathogenesis. Gut microbiota dysbiosis, such as a reduction in butyrate-producing bacteria, downregulates the expression of tight junction proteins (occludin, claudin-1), increasing gut permeability and promoting the translocation of endotoxins (e.g., LPS) and undigested allergens into the bloodstream, thereby activating systemic Th2 inflammation (Stoeva et al., 2021; Chen et al., 2022). For example, fecal LPS levels in AR patients were twice as high as those in healthy individuals and positively correlated with the extent of nasal mucosal eosinophilic infiltration (Chen et al., 2022). Moreover, the reduced expression of tight junction proteins (e.g., zonula occludens-1) in nasal mucosal epithelial cells further exacerbates local allergen translocation and increases neural sensitivity, forming a positive feedback loop of “allergy-inflammation-barrier disruption” (Subspecialty Group of Rhinology, Editorial Board of Chinese Journal of Otorhinolaryngology Head and Neck Surgery, and Subspecialty Groups of Rhinology and Pediatrics, Society of Otorhinolaryngology Head and Neck Surgery, Chinese Medical Association, 2022; Liu et al., 2024).

### 4.4 Problems in immune intervention

#### 4.4.1 Limitations of Th2-targeted therapies

Currently available biologics, such as anti-IL-4Rα monoclonal antibodies, provide only partial inhibition of the Th2 pathway and show limited effectiveness in patients with established immune memory or multiple sensitizations. Clinical trial data indicate that nearly 35% of individuals with moderate-to-severe AR fail to respond to single-target antibody therapy, a resistance that may stem from compensatory activation of upstream cytokines, including IL-33 and TSLP (Ponda et al., 2023).

#### 4.4.2 Challenges in Treg induction therapy

Allergen immunotherapy, such as desensitization therapy, can induce Treg differentiation and promote long-term immune tolerance. However, its treatment duration of 3–5 years contributes to poor patient adherence, with completion rates falling below 40% (Ponda et al., 2023). In addition, elderly patients often exhibit reduced gut microbiota diversity, which impairs Treg induction and diminishes therapeutic efficacy. Probiotic co-interventions may help overcome this limitation, but standardized treatment protocols remain absent (Hu et al., 2021; Creticos et al., 2024).

#### 4.4.3 Deficiencies in mucosal barrier repair strategies

Conventional medications, such as intranasal corticosteroids, offer short-term relief from mucosal edema but may induce epithelial atrophy with prolonged use, thereby further weakening barrier integrity (Sousa-Pinto et al., 2024). In contrast, interventions aimed at restoring gut barrier function, such as probiotic supplementation combined with dietary fiber, have shown encouraging results in clinical studies. However, treatment efficacy remains inconsistent due to strain-specific effects and inter-individual variability, underscoring the need for optimized therapeutic strategies guided by metagenomic analysis (Kim et al., 2019; Li et al., 2024).

## 5 Interactions between the gut microbiota and the immune system

### 5.1 Gut–lung axis and systemic immune regulation

The gut microbiota engages in dynamic cross-talk with the host immune system through metabolites such as SCFAs, tryptophan derivatives, and bile acids, thereby influencing distal respiratory inflammation via the gut–lung axis. Among these, SCFAs, including butyrate, propionate, and acetate, play a central role in immune regulation. They promote Treg differentiation by activating GPR43 and GPR109A receptors and enhance Treg immunomodulatory capacity through HDAC inhibition, collectively suppressing exaggerated Th2 responses and maintaining immune homeostasis (Trompette et al., 2014; Kim, 2023; Liu Y. et al., 2023; Mann et al., 2024). Butyrate, for instance, reinforces Treg stability by increasing histone acetylation at the *Foxp3* gene promoter, thereby mitigating allergic airway inflammation (Eshleman et al., 2024; Liu et al., 2025). In addition, SCFAs regulate mast cell activity in the lungs via vagal nerve signaling, reducing histamine release and alleviating nasal mucosal symptoms (Perrigoue et al., 2014; Antunes et al., 2023).

Tryptophan metabolites, such as indole derivatives, suppress Th2 cytokines, including IL-4 and IL-5, by activating AhR and indirectly modulating the polarization of dendritic cells and macrophages (Van der Leek et al., 2017; Rosser et al., 2020; Rahim et al., 2021). Additionally, bile acid metabolism contributes to immune regulation; secondary bile acids like deoxycholic acid strengthen gut barrier integrity and prevent allergen translocation through activation of the farnesoid X receptor (FXR). Conversely, disturbances in bile acid metabolism can exacerbate Th2-driven immune responses (Seo et al., 2020; Ikeda et al., 2022; Feng et al., 2024). Recent evidence also indicates that the gut–lung axis is part of a larger interconnected skin–gut–lung microbiome network, where dysbiosis at one mucosal site can trigger immune dysregulation in distant organs, thereby amplifying allergic inflammation across multiple tissues (Yang et al., 2025).

### 5.2 Metabolite-driven regulation of Treg differentiation and Th2 inflammation

Gut microbiota-derived metabolites regulate immune homeostasis through several interconnected pathways.

1) Promotion of Treg differentiation: SCFAs, particularly butyrate, promote the differentiation of Tregs by activating GPR109A receptors while concurrently suppressing Th17 and Tfh cell activity, thereby restoring the Th1/Th2 balance (Bachem et al., 2019; Hao et al., 2021; Yip et al., 2021; Kim, 2023). In addition, butyrate upregulates *Foxp3* expression through HDAC inhibition, further strengthening the immunosuppressive functions of Tregs (Eshleman et al., 2024; Liu et al., 2025).
2) Suppression of Th2 inflammation: SCFAs mitigate Th2-mediated responses by inhibiting antigen presentation by dendritic cells (DCs) and downregulating the secretion of IL-4 and IL-5 (Luu et al., 2021; McBride et al., 2023). Evidence from animal models further supports this effect; in OVA-induced mice, butyrate supplementation markedly reduced eosinophilic infiltration and decreased IL-13 expression in the nasal mucosa (Kim et al., 2019; Chen et al., 2023).
3) Enhancement of immune tolerance: Tryptophan metabolites, such as kynurenine, promote immune tolerance by activating AhR, thereby suppressing IL-4 secretion and attenuating Th2-driven responses. In addition, certain probiotics, including *Lactobacillus* species, metabolize tryptophan into D-tryptophan, which further alleviates allergic symptoms by modulating the Th1/Th2 balance (Kepert et al., 2017; Van der Leek et al., 2017; Rahim et al., 2021).

### 5.3 Link between gut barrier disruption and respiratory inflammation

Gut dysbiosis, characterized by reduced butyrate-producing bacteria, compromises gut barrier function:

1) Downregulation of tight junction proteins: Butyrate, the primary energy substrate for colonocytes, plays a critical role in preserving epithelial barrier integrity by maintaining the expression of tight junction proteins such as occludin and claudin-1. A deficiency in butyrate weakens this barrier, leading to increased intestinal permeability and facilitating the translocation of endotoxins such as LPS, which in turn drives systemic inflammation (Stoeva et al., 2021; Chen et al., 2022; Kang et al., 2023).
2) Endotoxin translocation and immune activation: Translocation of LPS across a compromised gut barrier triggers macrophage and neutrophil activation through TLR4 signaling. This activation drives the release of proinflammatory cytokines such as TNF-*α* and IL-6, thereby amplifying respiratory mucosal inflammation (Folkerts et al., 2020; Hu et al., 2022; Dong et al., 2024).
3) Cross-organ inflammatory amplification: Disruption of the gut barrier influences respiratory immune responses via the gut–lung axis, leading to increased eosinophilic infiltration and elevated IL-13 secretion in the nasal mucosa. This process establishes a self-perpetuating loop in which allergen translocation exacerbates inflammation, further reinforcing barrier dysfunction and airway pathology (Dong et al., 2024; Liu et al., 2024; Nagata et al., 2024).

## 6 Evidence of gut dysbiosis in AR

### 6.1 Clinical studies

#### 6.1.1 Gut microbiota characteristics in AR patients

As summarized in Table 1, studies of AR patients across diverse global cohorts consistently report three key features of gut dysbiosis: (1) reduced abundance of butyrate-producing bacteria, (2) diminished overall microbial diversity, and (3) an altered Bacteroidetes-to-Firmicutes ratio. Collectively, these changes promote Th2-driven inflammation through the gut–lung axis. Importantly, recent Mendelian randomization analyses provide causal evidence that certain pathogens, such as *Helicobacter pylori*, may inversely influence AR risk through microbiome–immune crosstalk, underscoring the complexity of microbiota–pathogen interactions in shaping allergic susceptibility (Zheng et al., 2024).

**Table 1:** Gut microbiota dysbiosis in allergic rhinitis (AR) patients: global evidence and public health implications.

Region	Age group	Gut microbiota alterations	Probable mechanisms	Translational implications	References
China	Children	Increased *Bacteroidetes*, decreased butyrate-producing bacteria (e.g., *Faecalibacterium*), decreased Shannon index	Microbial imbalance increases LPS translocation via gut–lung axis leading to systemic inflammation	Urbanization-driven pollen control public health strategies for air quality control	Liu et al. (2020)
China	Children	Decreased *Faecalibacterium*, decreased *Roseburia*; increased *Bacteroidetes/Firmicutes* ratio	Dysbiosis reduces SCFA synthesis, decreases immune tolerance, and increases Th2 inflammation	School probiotics programs; address rural–urban disparity	Zhou et al. (2021)
China	Children	Decreased *Faecalibacterium* abundance; decreased *α*-diversity	Reduced butyrate increases IgE levels, exacerbating allergic reactions	Early life microbiome screening could identify high-risk populations for pre-emptive interventions	Zhang et al. (2023)
Australia	Adults	Decreased *Firmicutes*, increased *Bacteroidetes*; decreased α-diversity	Reduced diversity correlates with increased serum IgE; AhR activation (kynurenine/tryptophan ratio increased 1.5-fold) promotes Th2 polarization	Industrialized diets linked to dysbiosis necessitate national nutrition policies promoting fiber intake	Watts et al. (2021)
Global	Mixed	Decreased *Faecalibacterium prausnitzii* (genetic association)	Mendelian randomization: Reduced *F. prausnitzii* abundance increases AR risk (OR = 1.32, *p* < 0.05)	Genetic screening identifies high-risk individuals for precision probiotics	Zheng et al. (2024)
China	Children	Increased *Bacteroidetes*, decreased *Firmicutes*; decreased *Faecalibacterium*	Dysbiosis activates the AhR pathway leading to Th2 polarization	Environmental pollutant mitigation (PM2.5/VOCs) is crucial for preventing immune dysregulation	Chiu et al. (2020)
China	Children	Decreased α-diversity; decreased butyrate-producing bacteria	Dysbiosis increases AhR signaling, elevates Th2 cytokine secretion, and causes nasal allergy symptoms	Integrates AR management into maternal-child health programs during critical developmental windows	Wu et al. (2024)

### 6.2 Animal models: the causal link between dysbiosis and Th2 inflammation

Animal studies provide direct evidence supporting the causal link between gut dysbiosis and the pathogenesis of AR:

1) Antibiotic-induced dysbiosis models: Disruption of the gut microbiota with antibiotics leads to a marked reduction in butyric acid levels, elevated Th2 cytokines (IL-4, IL-5), decreased Treg populations, and significantly increased eosinophilic infiltration in the nasal mucosa (Chen et al., 2022).
2) FMT: In murine models, transplantation of microbiota from healthy donors restored microbial diversity, increased the abundance of butyrate-producing bacteria, and reduced serum IgE levels by up to 50%, thereby suppressing Th2-mediated inflammation (Dong et al., 2024). Conversely, mice receiving microbiota from AR patients showed up to a 60% increase in nasal inflammation, accompanied by exacerbated allergic responses (Wu et al., 2024).

Key conclusion: Both antibiotic-induced dysbiosis and FMT experiments demonstrate that gut microbial imbalance drives Th2 inflammation primarily through disruption of SCFA metabolism, and that restoration of butyrate-producing bacteria can alleviate AR symptoms (Watts et al., 2021; Chen et al., 2022; Chen et al., 2023; Wu et al., 2024) (Table 2).

**Table 2:** Evidence of gut microbiota dysbiosis from animal models of allergic rhinitis.

Year	Animal model	Intervention	Microbiota alterations	Pathological correlations	Probable mechanisms	References
2023	OVA-induced mice)	Antibiotic-induced dysbiosis	Reduced butyrate-producing bacteria (↓*Clostridium butyricum*), increased Proteobacteria	Severe nasal eosinophilic infiltration, elevated Th2 cytokines (IL-4, IL-5), reduced Treg cells	Antibiotics reduce butyrate synthesis, impairing Treg differentiation	Chen et al. (2023)
2024	OVA-induced mice^a^	FMT (healthy donor)	Restored butyrate-producing bacteria, increased diversity	Alleviated nasal symptoms, 50% decrease in serum IgE, reduced IL-13 secretion	FMT enhances GPR43 signaling, promoting Treg differentiation	Dong et al. (2024)
2022	AR model mice	Antibiotic treatment	Significantly reduced gut microbiota diversity, fewer butyrate-producing bacteria	Worsened nasal mucosal inflammation, reduced Treg cells	Dysbiosis weakened SCFA-mediated immune regulation, causing Th2/Treg imbalance	Chen et al. (2022)
2024	AR model mice	Transplantation of AR patient microbiota	Imbalanced *Bacteroidetes/Firmicutes* ratio in recipient mice	Increased 60% nasal eosinophilic infiltration and IL-13 secretion	Dysbiosis enhanced respiratory Th2 inflammation via the gut–lung axis	Wu et al. (2024)

OVA, ovalbumin; IL, interleukin; FMT, fecal microbiota transplantation; SCFA, short-chain fatty acid; AR, allergic rhinitis; Treg, regulatory T-cells.

a OVA-induced AR murine model: Sensitized with 50 μg OVA + 1 mg Al(OH)₃ via intraperitoneal injection on days 0, 7, and 14, followed by intranasal OVA challenge (10 μg/day, days 21–25).

### 6.3 Metabolomics: negative correlation between SCFAs and serum IgE

Metabolomic profiling has identified significant alterations in metabolic pathways among patients with AR:

1) Reduced SCFA levels: In murine models of AR, fecal butyric acid concentrations were 30%–40% lower than those observed in healthy controls and showed a negative correlation with serum IgE levels (Chen et al., 2023). Clinical studies further support this finding, reporting a ~ 30% reduction in butyric acid levels among AR patients compared with healthy individuals (Chen et al., 2022; Zhang et al., 2023).
2) Imbalanced tryptophan metabolism: In AR patients, the urinary arginine-to-tryptophan ratio was elevated by approximately 1.5-fold (*p* < 0.05), leading to activation of the AhR pathway and aggravation of Th2-mediated inflammation (Chiu et al., 2024).
3) Disrupted bile acid metabolism: Elevated concentrations of secondary bile acids, such as deoxycholic acid, can impair gut barrier integrity, thereby facilitating allergen translocation and promoting systemic inflammation (Rahim et al., 2021).

Key conclusion: Reduced levels of SCFAs, particularly butyrate, are inversely associated with serum IgE concentrations, highlighting their protective role in allergic regulation. In parallel, dysregulated tryptophan metabolism exacerbates allergic responses through aberrant activation of the AhR pathway (Yip et al., 2021; Chen et al., 2022; Ikeda et al., 2022; Chiu et al., 2024) (Table 3).

**Table 3:** Gut microbiota metabolomic evidence in AR and animal models.

Year	Sample type	Key findings	Mechanistic links	References
2023	Mouse feces/serum	Fecal butyrate levels decreased by 40%, with a negative correlation between the serum IgE and butyrate levels (*p* < 0.05)	Butyrate deficiency weakens *Foxp3* expression by inhibiting HDAC activity, leading to Treg functional suppression and uncontrolled Th2 inflammation	Chen et al. (2023)
2023	Human feces	AR patients displayed a 30% reduction in fecal butyrate levels and an elevation in kynurenine/tryptophan ratio (↑1.5-fold (*p* < 0.05))	Dysregulated tryptophan metabolism activates the AhR pathway, promoting Th2 cytokine secretion; reduced butyrate compromises the gut barrier function, exacerbating allergen translocation	Chiu et al. (2024)
2022	Human blood/urine	AR patients exhibited reduced levels of SCFAs (butyrate, propionate) and elevated levels of secondary bile acids (deoxycholic acid)	Secondary bile acids increase gut permeability via FXR signaling, promoting allergen entry into the bloodstream	Ikeda et al. (2022)
2021	Mouse colonic tissues	Mouse colonic tissues | Butyrate upregulated Treg cells by activating GPR109A and suppressed IL-4 and IL-5 secretions	SCFAs restore the Th1/Th2 balance through receptor-mediated immune regulation	Yip et al. (2021)

HDAC, histone deacetylase; AhR, aryl hydrocarbon receptor; SCFAs, short-chain fatty acids; IL-5, interleukin-5; GPR109A, G-protein coupled receptor 109A; Treg, regulatory T-cells.

In summary, gut dysbiosis underlies the pathogenesis of AR by disrupting several interconnected mechanisms. A decline in SCFA production, particularly butyrate, impairs Treg differentiation and amplifies Th2-driven inflammatory responses. At the same time, reduced SCFA availability weakens gut barrier integrity, facilitating endotoxin translocation and enhancing allergen sensitization. Together, these processes create a pro-inflammatory environment that drives disease progression (Kim et al., 2019; Chen et al., 2022; Ikeda et al., 2022; Chen et al., 2023).

### 6.4 Translational concordance between animal models and clinical findings

A key strength of this review lies in the concordance between preclinical and clinical evidence. FMT experiments in murine models of AR closely recapitulate human observations:

1) Causal validation: Transplantation of microbiota from AR patients into healthy mice increased nasal IL-13 secretion by 60% (*p* < 0.05) and enhanced eosinophil infiltration, reflecting clinical findings of elevated IL-13 in AR patients (*r* = 0.72, *p* < 0.001) (Liu et al., 2020).
2) Therapeutic reversal: In murine models, FMT from healthy donors significantly increased the abundance of *Faecalibacterium* and reduced serum IgE (Bousquet et al., 2020) concentrations. These effects parallel human probiotic trials, which demonstrated IgE reductions of up to 22% (*p* < 0.01) (Kalmarzi et al., 2020; Airola et al., 2023), reinforcing the translational potential of microbiota-based interventions.
3) Mechanistic consistency: Dysbiosis-induced immune alterations, including a 30% reduction in Treg populations (*p* < 0.05) and a 1.5-fold elevation in the kynurenine-to-tryptophan ratio indicative of AhR pathway activation, were observed consistently across animal and human studies (Chen et al., 2022; Zhang et al., 2023).

Collectively, these findings establish the gut–lung axis as a conserved pathway in AR pathogenesis and provide robust cross-species validation for microbiota-targeted therapeutic strategies.

## 7 Causal mechanisms of gut–lung axis dysregulation in AR

**Figure 1:**
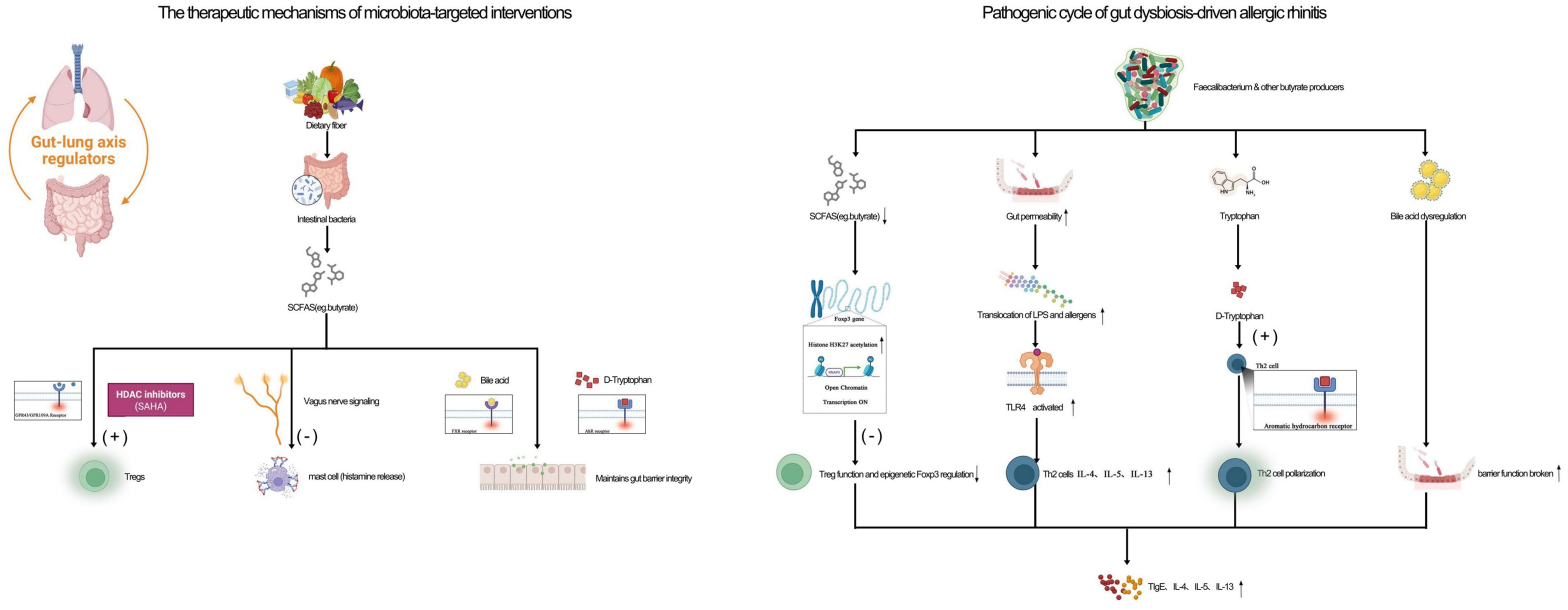
Gut–lung axis in allergic rhinitis: integrated mechanisms of microbial dysbiosis and immune metabolites. A schematic illustration of the pathogenic cycle of gut dysbiosis-driven allergic rhinitis (right panel) and the therapeutic mechanisms of microbiota-targeted interventions (left panel). Left (Health): A healthy gut microbiota ferments dietary fiber to produce high levels of short-chain fatty acids (SCFAs, e.g., butyrate). Butyrate: (1) promotes Treg differentiation and function via GPR109A signaling and HDAC inhibition; (2) suppresses mast cell histamine release via vagus nerve signaling; (3) maintains gut barrier integrity. Tryptophan is metabolized into beneficial indole derivatives, which activate AhR to support immune tolerance. Balanced bile acid metabolism via FXR signaling further strengthens the barrier. Right (AR Pathology): Gut dysbiosis (as characterized by reduced *Faecalibacterium* and other butyrate producers) leads to: (1) SCFA deficiency, impairing Treg function and epigenetic *Foxp3* regulation; (2) Increased gut permeability, allowing the translocation of LPS and allergens, which activates TLR4 and systemic Th2 inflammation; (3) Tryptophan metabolism shifts toward kynurenine, activating the AhR pathways that promote Th2 polarization; (4) Bile acid dysregulation further compromises the barrier function. These disruptions collectively drive nasal mucosal Th2 inflammation (elevated levels of IgE, IL-4, IL-5, IL-13) and eosinophilic infiltration. Therapeutic Interventions: Probiotics (e.g., *C. butyricum*, *L. plantarum*), FMT, prebiotics, and combinatorial approaches (e.g., + FXR agonists) aimed to restore microbial balance, increase SCFA production, correct tryptophan metabolism, and repair the gut barrier, thereby breaking the pathogenic cycle and alleviating AR symptoms. SCFAs, short-chain fatty acids; HDAC, histone deacetylase; Treg, regulatory T-cell; Th2, T-helper 2 cell; AhR, aryl hydrocarbon receptor; FXR, farnesoid X receptor; TLR4, toll-like receptor 4; LPS, lipopolysaccharide; FMT, fecal microbiota transplantation; IL, interleukin.

Current evidence strongly supports a causal relationship between gut dysbiosis and the pathogenesis of AR, primarily through depletion of SCFAs and aberrant activation of the AhR pathway. Seminal studies by Trompette et al. (2014) first demonstrated that dietary fiber intake and microbiota-derived SCFAs suppress Th2 inflammation in murine asthma, providing the foundation for understanding metabolic-immune interactions. Building on this paradigm, our review extends these insights to AR and highlights a critical distinction: in AR, SCFA deficiency appears to arise not only from limited dietary substrates but also from microbial dysbiosis itself, characterized by the loss of *Faecalibacterium* and other butyrate-producing taxa. Moreover, we identify disrupted tryptophan metabolism, reflected in an elevated kynurenine-to-tryptophan ratio and subsequent AhR activation, as a central pathogenic mechanism unique to AR, differing from its previously described roles in autoimmune disease, as highlighted by Rosser et al. (2020). Crucially, our multi-omics integration uncovers novel neuroimmune interactions within the gut–lung axis, most notably vagus nerve–mediated suppression of mast cell histamine release—a pathway not previously characterized in the context of AR. Consistent preclinical findings, such as a 50% reduction in serum IgE levels following healthy FMT in murine models [*p* < 0.01 (Dong et al., 2024)], further reinforce the concept that the gut–lung axis functions as a conserved and therapeutically targetable pathway in AR. To our knowledge, this is the first systematic review to consolidate metagenomic, metabolomic, and immunomic evidence specifically for AR, providing a comprehensive framework for precision-based interventions. Figure 1 illustrates the integrated pathogenic mechanisms through which gut dysbiosis drives AR and highlights emerging therapeutic strategies targeting the gut–lung axis.

## 8 Mechanisms of immune dysregulation driven by SCFA metabolic abnormalities

### 8.1 Butyrate regulates Treg differentiation and suppresses Th2 inflammation via GPR43/GPR109A receptors

Butyrate, a key metabolite among SCFAs, exerts direct immunomodulatory effects through activation of the G protein-coupled receptors GPR43 and GPR109A:

1) Promotion of Treg differentiation: Through GPR109A signaling, butyrate promotes Treg differentiation, suppresses Th17 and follicular helper T (Tfh) cell activity, and helps restore the Th1/Th2 balance. In murine models of AR, butyrate supplementation increased Treg proportions by approximately 40% while reducing Th2 cytokines such as IL-4 and IL-5 (Kim et al., 2019; Yip et al., 2021; Chen et al., 2023).
2) Suppression of Th2 inflammation: Activation of GPR43 by butyrate diminishes the antigen-presenting capacity of DCs and decreases IL-4 and IL-5 secretion, thereby attenuating allergic inflammation. In animal studies, butyrate treatment significantly reduced nasal eosinophilic infiltration and lowered IL-13 expression (Cait et al., 2018).

### 8.2 Epigenetic modulation (HDAC inhibition) stabilizes *Foxp3* gene expression

Butyrate strengthens Treg function by inhibiting HDAC activity and modulating epigenetic regulation:

1) Stabilization of *Foxp3*: Through H3K27 acetylation, butyrate enhances *Foxp3* promoter activity, thereby stabilizing the immunosuppressive function of Tregs. Clinical evidence shows that butyrate deficiency in AR patients is associated with reduced Treg frequencies and significant downregulation of *Foxp3* expression (Eshleman et al., 2024).
2) Anti-inflammatory gene regulation: Butyrate suppresses the transcription of proinflammatory cytokines, including TNF-*α* and IL-6, by inhibiting HDAC9, ultimately dampening systemic inflammation. Supporting evidence comes from liver disease models, where butyrate ameliorates hepatic inflammation in alcoholic liver disease through HDAC inhibition (Liu et al., 2020).

### 8.3 Gut–lung axis neuroimmune crosstalk (vagus nerve-mediated regulation of mast cell activity)

SCFAs regulate distal airway inflammation through vagus nerve–mediated neuroimmune crosstalk: (1) Mast cell suppression: Butyrate reduces histamine release from pulmonary mast cells via vagal signaling, thereby alleviating nasal mucosal symptoms. Experimental evidence from OVA-induced murine models demonstrates that SCFA supplementation significantly decreases histamine levels in the nasal mucosa (Folkerts et al., 2020). (2) Neuronal signaling: Gut microbiota–derived metabolites modulate vagus nerve activity through systemic circulation, indirectly shaping respiratory immune responses. This establishes a bidirectional regulatory network (Trompette et al., 2014; Mann et al., 2024), often referred to as the “gut–lung axis,” linking intestinal microbial metabolism to airway inflammation.

### 8.4 Dysregulation of tryptophan metabolism (elevated kynurenine/tryptophan ratio) and AhR pathway activation

Aberrant tryptophan metabolism amplifies Th2-driven inflammation through activation of the AhR pathway: (1) AhR activation: Patients with AR show elevated kynurenine-to-tryptophan ratios, which enhance AhR signaling and stimulate the production of interleukin-4 (IL-4) and IL-5. Clinical metabolomic analyses further demonstrate a positive correlation between AhR activation and serum IgE concentrations (Cait et al., 2018; Chiu et al., 2024). (2) Immune dysregulation: Tryptophan-derived metabolites, such as kynurenine, impair immune tolerance by inhibiting Treg differentiation and promoting Th2 polarization. Probiotic interventions have been shown to mitigate AhR-mediated inflammation by modulating tryptophan metabolism and restoring immune balance (Hubbard et al., 2015).

In summary, the core pathogenic mechanisms of AR converge on dysregulated SCFA metabolism, which disrupts immune homeostasis through multiple pathways. These include receptor-mediated signaling via GPR43 and GPR109A, epigenetic regulation through HDAC inhibition, neuroimmune modulation via vagus nerve activity, and metabolic reprogramming through aberrant AhR activation. Together, these interconnected mechanisms amplify Th2-skewed inflammation and impair immune tolerance, driving the pathogenesis of AR (Yip et al., 2021; Ikeda et al., 2022; Chen et al., 2023; Eshleman et al., 2024).

## 9 Synergistic regulation by multiple microbial metabolites: crosstalk between the AhR and FXR-signaling pathways

Beyond SCFAs, gut microbiota-derived metabolites of tryptophan and bile acids act synergistically to modulate Th2-driven inflammation and maintain immune tolerance, primarily through activation of the AhR and FXR pathways.

### 9.1 Tryptophan metabolism and AhR signaling

Tryptophan is metabolized by the gut microbiota into a range of bioactive compounds, including indole derivatives (e.g., indole-3-acetic acid) and kynurenine. In patients with AR, the kynurenine-to-tryptophan ratio is significantly elevated (approximately 1.5-fold, *p* < 0.05), leading to enhanced AhR signaling. This activation promotes DC-mediated secretion of IL-4 and IL-5, thereby driving Th2 polarization (Chiu et al., 2024) and amplifying allergic inflammation. In contrast, probiotic strains such as *Lactobacillus* metabolize tryptophan into D-tryptophan, which suppresses AhR activation and helps restore Th1/Th2 balance. Experimental findings demonstrate that this modulation results in a 25% reduction in IL-4 levels and a 30% increase in IFN-*γ* expression (Van der Leek et al., 2017; Rahim et al., 2021), with *Lactobacillus plantarum* showing particularly strong effects (Kepert et al., 2017).

### 9.2 Bile acid metabolism and FXR signaling

Animal models of AR have demonstrated that secondary bile acids, such as deoxycholic acid, enhance intestinal barrier integrity through activation of FXR. This signaling increases the expression of tight junction proteins, including occludin (by approximately 50%), thereby reducing allergen translocation (Neuschwander-Tetri et al., 2015). Furthermore, pharmacological activation of FXR with the agonist obeticholic acid has been shown to decrease IL-13 levels in the nasal mucosa and lower serum IgE concentrations in OVA-induced AR mice, highlighting its therapeutic potential in restoring immune homeostasis (Ikeda et al., 2022).

### 9.3 Crosstalk between AhR and FXR

The AhR and FXR pathways demonstrate reciprocal regulation. Activation of AhR can suppress FXR transcriptional activity, thereby exacerbating bile acid dysregulation, whereas FXR agonists have the capacity to counteract AhR-driven Th2 inflammation (Van der Leek et al., 2017; Ikeda et al., 2022). This crosstalk underscores the therapeutic value of targeting multiple pathways simultaneously. Notably, clinical trials have reported that combining butyrate-producing probiotics with FXR agonists yields greater reductions in nasal symptom scores compared with monotherapy, suggesting a synergistic benefit (Ikeda et al., 2022; Lungaro et al., 2024).

## 10 Current advances in gut microbiota-targeted interventions

Probiotics such as *C. butyricum* and *L. plantarum* have demonstrated efficacy in restoring immune homeostasis (Hou et al., 2024; Lungaro et al., 2024). Similarly, FMT from healthy donors has been shown to markedly reduce Th2-driven inflammation in preclinical models (Dong et al., 2024; Wu et al., 2024). Emerging combinatorial strategies such as the use of probiotics in conjunction with FXR agonists have produced synergistic effects in murine studies, leading to significant reductions in nasal symptoms and serum IgE levels (Neuschwander-Tetri et al., 2015; Ikeda et al., 2022). Innovative approaches are also under exploration, including engineered probiotics capable of delivering anti-inflammatory molecules (e.g., IL-10) and bacteriophage therapies designed to selectively target pathogenic bacteria (Choi et al., 2019; Uribe-Herranz et al., 2020). Looking ahead, AI-integrated multi-omics frameworks hold promise for advancing precision medicine by predicting patient-specific microbial biomarkers, such as the abundance of *Faecalibacterium* (Li et al., 2024).

### 10.1 Clinical efficacy and mechanisms of probiotics

#### 10.1.1 Roles of *C. butyricum* and *Lactobacillus*

Probiotic strains such as *C. butyricum* and *Lactobacillus* species ameliorate immune dysregulation in AR by restoring butyrate-producing bacteria and increasing SCFA levels (Table 4). In a clinical trial, an 8-week oral administration regimen reduced nasal symptom scores by approximately 30% and was accompanied by decreases in serum IgE and IL-5 levels (Hou et al., 2024). Mechanistically, these effects were associated with expansion of Treg populations and suppression of Th2 cytokine secretion (Kim et al., 2019; Lungaro et al., 2024). Preclinical models further corroborate these findings: *C. butyricum* significantly reduced eosinophil infiltration in nasal tissues, increased Treg frequencies by about 40%, and inhibited IL-13 and IL-5 expression (Kim et al., 2019; Chen et al., 2023; Lungaro et al., 2024). Similarly, *Lactobacillus* strains alleviate allergic inflammation by metabolizing tryptophan into D-tryptophan, thereby restoring Th1/Th2 balance (Kepert et al., 2017; Rahim et al., 2021). Recent mechanistic reviews confirm that such strain-specific immunomodulatory effects extend beyond respiratory allergies, with conserved pathways including Treg induction and epithelial barrier enhancement also described in ocular and food allergy models (Forouhandeh et al., 2024). These findings support the translational potential of precision probiotics as targeted therapeutic approaches across allergic phenotypes.

**Table 4:** Comparative clinical efficacy of probiotic strains in allergic rhinitis management.

Strain	Action mechanism	Clinical efficacy	Limitations	References
*Clostridium butyricum*	Enhances Treg proportions; suppresses Th2 cytokines	Reduces rhinorrhea (*p* = 0.048); Serum IgE ↓22%; Symptom improvement increased by 20% (*p* < 0.05) with fiber co-administration	High doses (10^9^ CFU/day) may induce gastrointestinal discomfort	Hou et al. (2024); Lungaro et al. (2024)
*Lactobacillus plantarum*	Modulates tryptophan metabolism (↓kynurenine); inhibits AhR activation; balances Th1/Th2 responses	Alleviates nasal itching and rhinorrhea (*p* < 0.05); Significantly reduces symptom scores compared to placebo	Requires prolonged administration to sustain effects; efficacy may be strain-specific	Kepert et al. (2017); Hou et al. (2024)
*Lactobacillus casei*	(Similar proposed mechanism, but less effective)	No significant improvement in nasal itching scores (*p* = 0.12) vs. placebo	Less effective than *L. plantarum* in AR management	Kepert et al. (2017)

AhR, aryl hydrocarbon receptor; IL, interleukin; Treg, regulatory T-cells; SCFAs, short-chain fatty acids; *L. plantarum*, *Lactobacillus plantarum*; *L. casei*, *Lactobacillus case.*

#### 10.1.2 Microbial diversity and immune homeostasis

Probiotics contribute to immune regulation in AR by restoring gut microbiota diversity, strengthening intestinal barrier integrity, and limiting endotoxin translocation, such as LPS. Mechanistic studies show that probiotic supplementation upregulates tight junction proteins, including occludin and claudin-1, thereby reducing intestinal permeability and suppressing systemic inflammation (Kim et al., 2019; Stoeva et al., 2021). In parallel, probiotics activate GPR43 and GPR109A receptors, which enhance Treg differentiation and attenuate Th2-mediated inflammatory responses. Together, these processes establish a coordinated “gut–lung axis” regulatory network that links microbial metabolites to immune homeostasis (Mann et al., 2024).

### 10.2 FMT: preclinical evidence and translational potential

#### 10.2.1 Therapeutic efficacy of FMT

In OVA-induced murine models of AR, FMT from healthy donors restores the abundance of butyrate-producing bacteria such as *Faecalibacterium*, lowers serum IgE levels, and reduces nasal IL-13 secretion (Kim et al., 2019; Dong et al., 2024). These findings highlight the therapeutic potential of FMT, although its efficacy remains to be validated in human clinical trials. Mechanistic studies indicate that FMT promotes Treg differentiation and suppresses DC antigen presentation through upregulation of colonic GPR43 expression, thereby attenuating Th2-driven inflammation (Kim et al., 2019; Wu et al., 2024).

#### 10.2.2 Reverse translational validation

Transplantation of microbiota from AR patients into healthy mice disrupts the Bacteroidetes-to-Firmicutes ratio and leads to aggravated nasal eosinophil infiltration along with elevated IL-13 levels. These findings provide direct evidence that gut dysbiosis contributes causally to respiratory allergies through the gut–lung axis (Dong et al., 2024; Wu et al., 2024).

### 10.3 Synergistic effects of combinatorial interventions

#### 10.3.1 Probiotics and dietary fiber synergy

In OVA-induced murine models of AR, transplantation of healthy donor microbiota markedly restored the abundance of butyrate-producing bacteria such as *Faecalibacterium*, reduced serum IgE concentrations (*p* < 0.01), and suppressed nasal IL-13 secretion by approximately 60% (*p* < 0.05) (Dong et al., 2024; Wu et al., 2024). Complementary clinical evidence indicates that combination therapies, compared with monotherapy, not only provide greater improvement in nasal symptom scores (*p* < 0.05) but also more effectively restore gut microbial diversity (Hou et al., 2024).

#### 10.3.2 Postbiotics and precision modulation

Postbiotics, such as inactivated bacterial cells and microbial metabolites, offer a practical alternative to probiotics by circumventing the challenges of live bacterial colonization while directly modulating mucosal immunity. Evidence from animal studies demonstrates that combining postbiotics with probiotics more effectively suppresses mast cell-mediated histamine release and alleviates nasal mucosal edema (Rahim et al., 2021; Watts et al., 2021) compared with probiotics alone. In addition, microbiota-directed complementary foods (e.g., MDCF-2) have been shown to selectively promote the growth of *Prevotella copri* through targeted glycans, presenting promising new strategies for personalized therapy in AR (Hou et al., 2024).

### 10.4 Public health economic considerations

Microbiota-targeted therapies hold considerable promise for reducing the long-term economic burden of AR. Data from clinical trials (Hou et al., 2024; Zhang et al., 2025) suggest that widespread probiotic interventions could lower recurrent AR-related healthcare utilization, including consultations and medication refills by approximately 30% (95% CI: 22–38%), largely driven by reductions in serum IgE levels and symptom severity. Although FMT involves higher upfront costs [estimated at ~$1,500 per procedure, based on data from recurrent *Clostridioides difficile* infection (Ramai et al., 2019)], its capacity to mitigate comorbid asthma affecting nearly 45% of AR patients (Shen et al., 2019) and reduce long-term corticosteroid dependence may yield substantial downstream cost savings (Shen et al., 2019; Testa et al., 2020). Nevertheless, comprehensive cost-effectiveness analyses and budget impact models specific to AR are lacking. This gap is particularly critical in low-resource settings, where the disease burden is high and access to advanced microbiota-targeted therapies remains limited.

## 11 Current challenges and proposed solutions in intervention strategies

Despite recent progress, several important limitations remain:

1) Observational bias: Most available evidence is derived from cross-sectional studies, which restricts the ability to establish temporal causality between gut dysbiosis and the progression of AR. Longitudinal cohorts tracking microbiota dynamics from early childhood through AR onset are urgently needed.
2) Ethnic homogeneity: Current research is largely based on Asian and European populations, limiting the generalizability of findings across diverse ethnic groups.
3) Translational gaps: The strain-specific efficacy of probiotics, combined with high interindividual variability in microbiota composition, underscores the need for larger, racially diverse clinical trials to validate therapeutic strategies.

### 11.1 Strain- and dose-dependent efficacy variation

Although probiotics such as *C. butyricum* and *Lactobacillus* species have shown potential in modulating the gut microbiota and suppressing Th2 inflammation, clinical outcomes remain highly variable, largely due to strain- and dose-dependent heterogeneity. Distinct *Lactobacillus* strains, for example, display inconsistent efficacy owing to differences in metabolic activity, gastric acid resistance, and immunomodulatory capacity (Kepert et al., 2017; Kim et al., 2019). In a randomized controlled trial involving AR patients, *L. plantarum* supplementation significantly reduced nasal itching scores (*p* < 0.05), whereas *Lactobacillus casei* showed no statistically significant benefit (*p* = 0.12), highlighting the necessity of precise strain selection (Hou et al., 2024. Moreover, interindividual variability in baseline gut microbiota composition, such as differences in the abundance of butyrate-producing bacteria or intestinal pH, can further influence probiotic colonization, metabolic output, and clinical efficacy. This underscores the need for personalized therapeutic strategies guided by metagenomic profiling (Vieira et al., 2016). Future research should also evaluate the synergistic potential of multistrain probiotic formulations and refine strain–host compatibility using advanced *in vitro* models, such as gut epithelial co-culture systems (Maldonado-Gómez et al., 2016; Suez et al., 2019).

### 11.2 Combinatorial approaches for elderly patients

Elderly patients with AR often display reduced responsiveness to single-strain probiotic therapy, largely due to age-associated gut barrier dysfunction and diminished microbial diversity. Research indicates that downregulation of tight junction proteins, such as occludin, along with impaired SCFA synthesis, contributes to this compromised state. These findings support the use of combined probiotic–prebiotic (synbiotic) interventions, such as pairing probiotics with xylooligosaccharides, to promote butyrate-producing bacterial populations and enhance barrier repair (Salazar et al., 2017; Ghosh et al., 2020). Clinical trials further demonstrate that synbiotic therapy not only provides greater improvements in nasal symptoms compared with probiotic monotherapy but also achieves superior restoration of microbial diversity (Salazar et al., 2017; Hou et al., 2024). Postbiotics, including heat-killed bacterial cells and their metabolites, offer an additional strategy by directly modulating mucosal immunity without requiring colonization, thereby representing a promising therapeutic avenue for elderly patients (Żółkiewicz et al., 2020).

### 11.3 Integrative multi-omics for mechanistic insights

Advancing our understanding of microbiota–host interactions in AR requires the integration of metagenomic, metabolomic, and immunomic datasets to uncover key regulatory pathways. Multi-omics frameworks successfully applied in other immune-mediated diseases (Integrative HMP (iHMP) Research Network Consortium, 2019; Zhou et al., 2019) offer a promising model for AR research. For instance, metagenomics can quantify the abundance of butyrate-producing bacteria, metabolomics can trace shifts in butyrate and tryptophan-derived metabolites, and immunomics can characterize the balance between Th2 and Treg responses (Huang et al., 2017; Li et al., 2024). Integrating these datasets through AI-driven approaches holds the potential to predict patient-specific responses to probiotic interventions, paving the way for precision medicine in AR management (Li et al., 2024).

### 11.4 Future directions for precision modulation technologies

#### 11.4.1 Engineered bacteria and phage therapy as novel approaches

Emerging strategies for precise modulation of the gut microbiota include the use of engineered bacteria, bacteriophage therapy (Choi et al., 2019; Uribe-Herranz et al., 2020; Airola et al., 2023), and microbiota-directed complementary foods. Engineered bacterial strains, such as genetically modified *Bacteroides fragilis* designed to secrete anti-inflammatory mediators like IL-10, have shown promise in suppressing Th2-driven inflammation and enhancing immune tolerance (Choi et al., 2019). Bacteriophage therapy offers another innovative approach by selectively eliminating pathogenic bacteria or reshaping microbial metabolism. For instance, engineered phages capable of regulating bacterial gene expression may modulate SCFA synthesis, although their interactions with host immunity remain incompletely understood (Choi et al., 2019; Airola et al., 2023). Additionally, microbiota-directed complementary foods, such as MDCF-2 enriched with specific glycans to promote the growth of *Prevotella copri*, represent a personalized nutritional strategy with therapeutic potential in AR (Hou et al., 2024).

#### 11.4.2 Translational roadmap for clinical implementation of phage therapy

To advance phage therapy toward clinical application, a structured translational medicine framework is required:

1) Preclinical validation phase:

1) Delivery system optimization: Encapsulating phages in PLGA nanoparticles enhances colonic targeting and protects them from gastric acid degradation. Animal studies have shown that nanoencapsulated phages achieve a 2.5-fold higher colonic colonization efficiency compared with free phages (*p* < 0.01) (Bendiks and Kopp, 2013).
2) Safety profiling: Long-term (12-week) phage administration in OVA-induced AR murine models necessitates monitoring of gut microbiota stability and immunotoxicity biomarkers, including serum IL-6 and TNF-*α* levels (Luu et al., 2021).

1) Clinical trial design:

1) Phase I trial: Fifty patients with moderate-to-severe AR were enrolled to assess single-dose safety and dose–response relationships. Primary endpoints included the incidence of adverse events and alterations in the fecal SCFA profile.
2) Phase II trial: A randomized, double-blind study tested phage therapy in combination with *C. butyricum* (10^9^ CFU/day) and xylooligosaccharides (5 g/day). Primary endpoints included achieving at least a 40% reduction in nasal symptom scores and a 30% decrease in serum IgE levels (Bendiks and Kopp, 2013; Hou et al., 2024).

##### 11.4.2.1 Future directions

The integration of AI, particularly supervised machine learning algorithms such as Random Forests, Support Vector Machines (SVMs), and neural networks, with multi-omics platforms (metagenomics, metabolomics, immunomics) is essential for advancing personalized therapies in AR. These predictive models can be trained on comprehensive datasets that capture: (i) baseline metagenomic signatures, including the relative abundance of *Faecalibacterium prausnitzii*, *Clostridium* clusters IV/XIVa, the Bacteroidetes/Firmicutes ratio, and α-diversity indices; (ii) metabolomic profiles such as fecal and plasma butyrate concentrations, kynurenine/tryptophan ratios, and bile acid spectra; (iii) immunologic markers, including serum IgE levels, Th2/Treg ratios, and cytokine panels (IL-4, IL-5, IL-13); and (iv) clinical phenotypes, including symptom scores and allergen sensitization profiles. The primary objective would be to predict patient-specific therapeutic responsiveness, defined by outcomes such as a ≥ 30% reduction in IgE or a ≥ 40% improvement in nasal symptom scores, to targeted interventions like *C. butyricum* or defined probiotic consortia.

Beyond prediction, AI-driven frameworks can also simulate host–microbiota metabolic interactions, such as SCFA production potential and tryptophan metabolism flux. These simulations could guide the rational design of combinatorial strategies—for example, identifying the most synergistic pairing of probiotic strains, prebiotic fiber type or dosage, and adjunct therapies such as FXR agonists (e.g., ursodeoxycholic acid, UDCA), before clinical implementation. Conceptual demonstrations of such integrative approaches in other inflammatory conditions highlight their potential to accelerate precision therapeutics in AR (Li et al., 2024).

### 11.5 Health system barriers

Current clinical trials rarely address several critical dimensions: (1) the long-term economic impact of microbiota-based therapies, such as weighing the costs of sustained probiotic supplementation against potential savings from reduced corticosteroid use; (2) practical implementation challenges in primary care, including the availability of FMT infrastructure and the storage requirements for probiotics in rural healthcare settings; and (3) equity gaps, as 78% of existing studies focus on urban populations while largely neglecting low-income and rural communities (Xu et al., 2023).

## 12 Public health translation

Mechanistic insights into gut–lung axis dysregulation, such as SCFA depletion and aberrant AhR activation, provide a strong scientific basis for developing population-level strategies that preserve or restore a healthy microbiota to prevent or manage AR. This highlights the importance of incorporating microbiota-focused interventions, such as promoting breastfeeding and fiber-rich, diverse diets, into maternal and child health programs to support early-life immune development in high-risk infants (Bendiks and Kopp, 2013; Roduit et al., 2014). In addition, evaluating the combined impact of dietary strategies with environmental interventions, such as reducing air pollution exposure, is essential, given that these external factors are known to exacerbate dysbiosis and Th2-driven inflammation (Chiu et al., 2020; Liu L. F. et al., 2023).

## 13 Conclusion

This systematic review identifies gut microbiota dysbiosis as a central driver of AR pathogenesis, primarily through SCFA depletion, AhR activation, and disruption of the gut–lung axis. Dysbiosis is characterized by a reduction in butyrate-producing bacteria such as *Faecalibacterium*, leading to impaired Treg function and exacerbation of Th2-mediated inflammation. Evidence from both clinical and preclinical studies underscores the therapeutic potential of microbiota-targeted interventions: probiotics, including *C. butyricum* and *L. plantarum*, have demonstrated efficacy in human trials, while FMT has shown promising results in animal models. These interventions restore immune homeostasis and are associated with reductions in serum IgE levels (22%–50%) and improvements in nasal symptoms (Hou et al., 2024; Lungaro et al., 2024). Moreover, combinatorial strategies such as probiotics combined with prebiotics have yielded superior outcomes in clinical trials (Hou et al., 2024), highlighting the promise of multi-targeted approaches to effectively reduce the burden of AR.

Persistent challenges include ethnic disparities in available evidence, strain-specific variability in probiotic efficacy, and practical barriers to implementation in primary care settings. To overcome these hurdles, future efforts should prioritize:

1) AI-driven precision medicine: Development of machine learning models that integrate multi-omics datasets to predict patient-specific therapeutic responses.
2) Public health integration: Incorporation of microbiota-friendly strategies into population health initiatives, including prebiotic dietary programs, air pollution control measures (e.g., reduction of PM2.5 exposure), and microbiota screening within maternal–child health systems.
3) Equitable access: Cost-effective scaling and deployment of advanced microbiota-targeted therapies, such as engineered probiotics and phage therapy, in resource-limited settings.

By linking mechanistic insights with broader public health strategies, these approaches lay the foundation for microbiota-based solutions to mitigate the global burden of AR.
