# Supplementary material for: Gut–lung axis in allergic rhinitis: microbial dysbiosis and therapeutic strategies
Source: Front Microbiol. 2025 Dec 12;16:1654997. doi: 10.3389/fmicb.2025.1654997 (PMC12742311; doi:10.3389/fmicb.2025.1654997)
Supplement: Supplementary file 1 [file Table_1.DOCX]

Supplementary Material

# Supplementary Tables

**Supplementary Table 1** Exclusion rationale for the assessed full-text articles.

| **Exclusion Category** | **Exclusion Reason** | **Examples of Excluded Studies (Title/Author/Year)** |
| --- | --- | --- |
| Non-allergic rhinitis studies | A focus on non-allergic rhinitis (e.g., non-allergic/vasomotor rhinitis) (n = 83) | 1. "Intranasal antihistamines in the treatment of idiopathic non-allergic rhinitis: a systematic review and meta-analysis" (Khoueir N, 2023)  2. "Surgical management of vasomotor rhinitis: a systematic review" (Halderman et al., 2015) |
| Small-sample studies | Small sample size (n < 20) or lack of a control group (n = 49) | 1. "Pilot Study on Allergic Rhinitis and Gut Microbiota" (Chen et al., 2022)  2. "Antihistamine Efficacy in AR: A Small Cohort" (Wang et al., 2019) |
| Incomplete outcome measures | Missing critical data (e.g., IgE levels, Treg/Th2 ratios) (n = 40) | 1. "Short-chain fatty acids in diseases" (Zhang et al., 2023)  2. "Microbiome and Allergic Diseases" (Garcia et al., 2021) |

Notes:

1. Exclusion Priority: Non-allergic rhinitis studies were prioritized for exclusion, followed by small-sample studies and those with incomplete outcomes.

2. Transparency: Representative examples of the excluded studies are provided above. The full list can be made available upon request to the corresponding author.

3. Alignment with PRISMA: This table ensures adherence to PRISMA guidelines by detailing the exclusion rationale and maintaining reproducibility.

**Supplementary Table 2** Distribution of global incidence of allergic rhinitis (AR).

| **Area** | **Crowd** | **Prevalence (%)** | **Key Risk Factors** | **References** |
| --- | --- | --- | --- | --- |
| China | Children | 10.8–21.1 | Urbanization-related pollen exposure variation | [3] |
| Middle East (Iran) | Children | 24.3 | Dust mites, air pollution | [13] |
| Europe (Poland) | Adult (LAR patients) | 17.6 | Allergen exposure, lifestyle changes, environmental factors, etc. | [14] |
| South Africa | Teenagers (13–14 years) | 20.2-25.4 | Urbanization, air pollution, allergen exposure, etc. | [63] |
