# Supplementary figures and images for: Gut–lung axis in allergic rhinitis: microbial dysbiosis and therapeutic strategies
Source: Front Microbiol. 2025 Dec 12;16:1654997. doi: 10.3389/fmicb.2025.1654997 (PMC12742311; doi:10.3389/fmicb.2025.1654997)

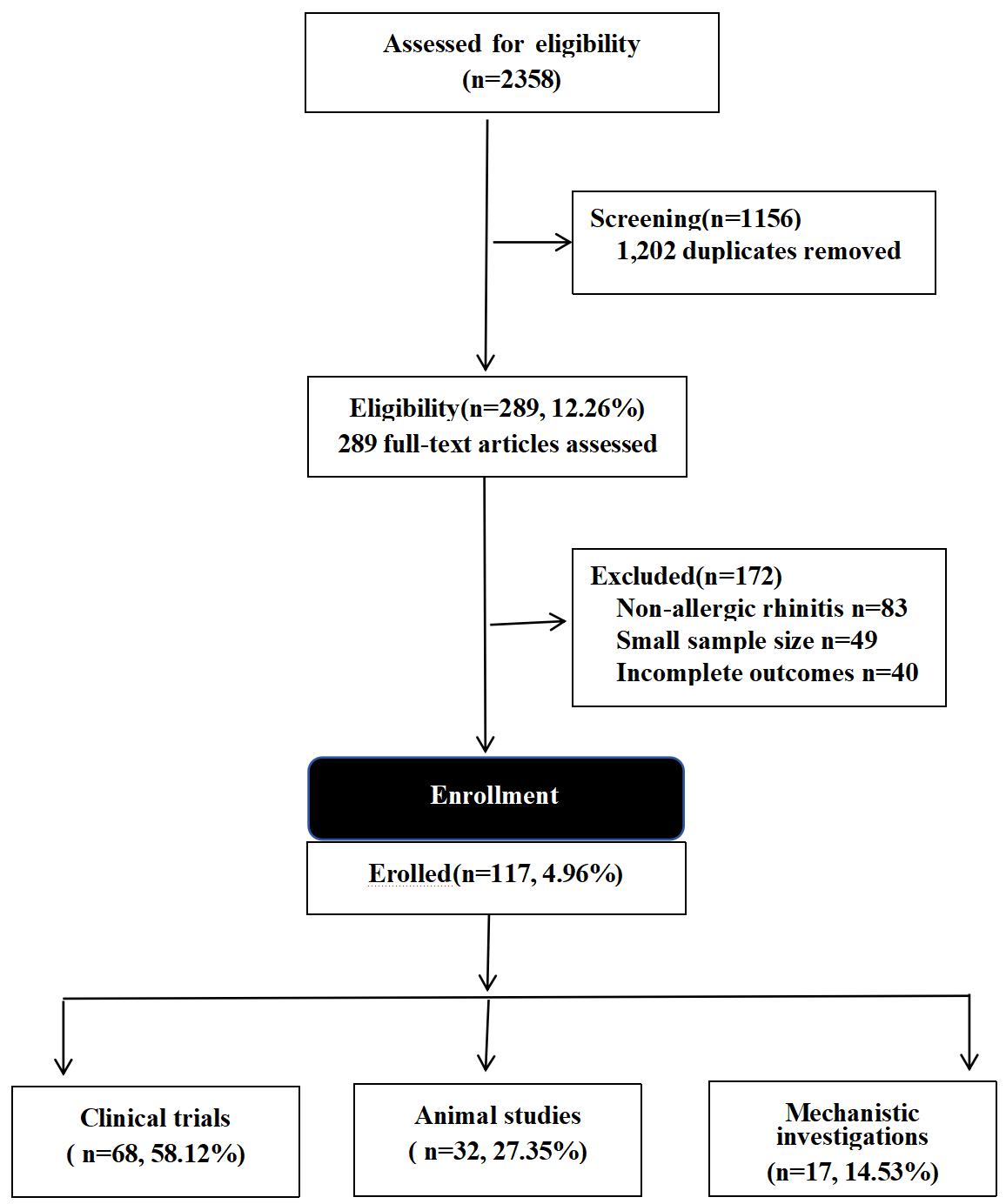

Supplement: Supplementary file 2 [file Image_1.JPEG]
